# Mitochondrial BK_Ca_ channel

**DOI:** 10.3389/fphys.2015.00104

**Published:** 2015-03-31

**Authors:** Enrique Balderas, Jin Zhang, Enrico Stefani, Ligia Toro

**Affiliations:** ^1^Department of Anesthesiology, University of California, Los AngelesLos Angeles, CA, USA; ^2^Deparment of Molecular and Medical Pharmacology, University of California, Los AngelesLos Angeles, CA, USA; ^3^Department of Physiology, University of California, Los AngelesLos Angeles, CA, USA; ^4^Brain Research Institute, University of California, Los AngelesLos Angeles, CA, USA; ^5^Cardiovascular Research Laboratory, University of California, Los AngelesLos Angeles, CA, USA

**Keywords:** mitochondria, potassium channels, BK channels, MaxiK channels, subunit composition, permeability transition pore, ischemia reperfusion injury

## Abstract

Since its discovery in a glioma cell line 15 years ago, mitochondrial BK_Ca_ channel (mitoBK_Ca_) has been studied in brain cells and cardiomyocytes sharing general biophysical properties such as high K^+^ conductance (~300 pS), voltage-dependency and Ca^2+^-sensitivity. Main advances in deciphering the molecular composition of mitoBK_Ca_ have included establishing that it is encoded by the *Kcnma1* gene, that a C-terminal splice insert confers mitoBK_Ca_ ability to be targeted to cardiac mitochondria, and evidence for its potential coassembly with β subunits. Notoriously, β1 subunit directly interacts with cytochrome c oxidase and mitoBK_Ca_ can be modulated by substrates of the respiratory chain. mitoBK_Ca_ channel has a central role in protecting the heart from ischemia, where pharmacological activation of the channel impacts the generation of reactive oxygen species and mitochondrial Ca^2+^ preventing cell death likely by impeding uncontrolled opening of the mitochondrial transition pore. Supporting this view, inhibition of mitoBK_Ca_ with Iberiotoxin, enhances cytochrome c release from glioma mitochondria. Many tantalizing questions remain open. Some of them are: how is mitoBK_Ca_ coupled to the respiratory chain? Does mitoBK_Ca_ play non-conduction roles in mitochondria physiology? Which are the functional partners of mitoBK_Ca_? What are the roles of mitoBK_Ca_ in other cell types? Answers to these questions are essential to define the impact of mitoBK_Ca_ channel in mitochondria biology and disease.

## Introduction

Mitochondria are key organelles defining cell fate and thus, much interest has developed in understanding the functional role of proteins present at its membranes. Mitochondria are shaped by an outer membrane, and an internal membrane that separates the intermembrane space and the matrix. The internal membrane is particularly important because it houses the respiratory chain protein complexes where reactive oxygen species and ATP are produced and also serves to delimit a Ca^2+^ storage space, making mitochondria not only producers of ATP but also regulators of Ca^2+^ and redox homeostasis. The list of proteins uncovered at the inner membrane keeps increasing, among them, K^+^ selective channels. A recent review by Szabo and Zoratti (2014) summarizes the evidence for the presence/role of: ATP-sensitive (K_ATP_), small-conductance calcium-activated (SK_Ca_), intermediate-conductance calcium-activated (IK_Ca_), large-conductance, voltage and calcium-activated (BK_Ca_), voltage-gated 1.3 (Kv1.3), two-pore domain acid-sensitive type 3 (TASK-3), and pH-sensitive K^+^channels. The subject of this review is the mammalian mitochondrial BK_Ca_ channel (mitoBK_Ca_) that has sparked much interest primarily because of its role in protecting the heart from ischemic insult, as first demonstrated by Xu et al. (2002).

## General design of BK_Ca_ channels

**Figure 1:**
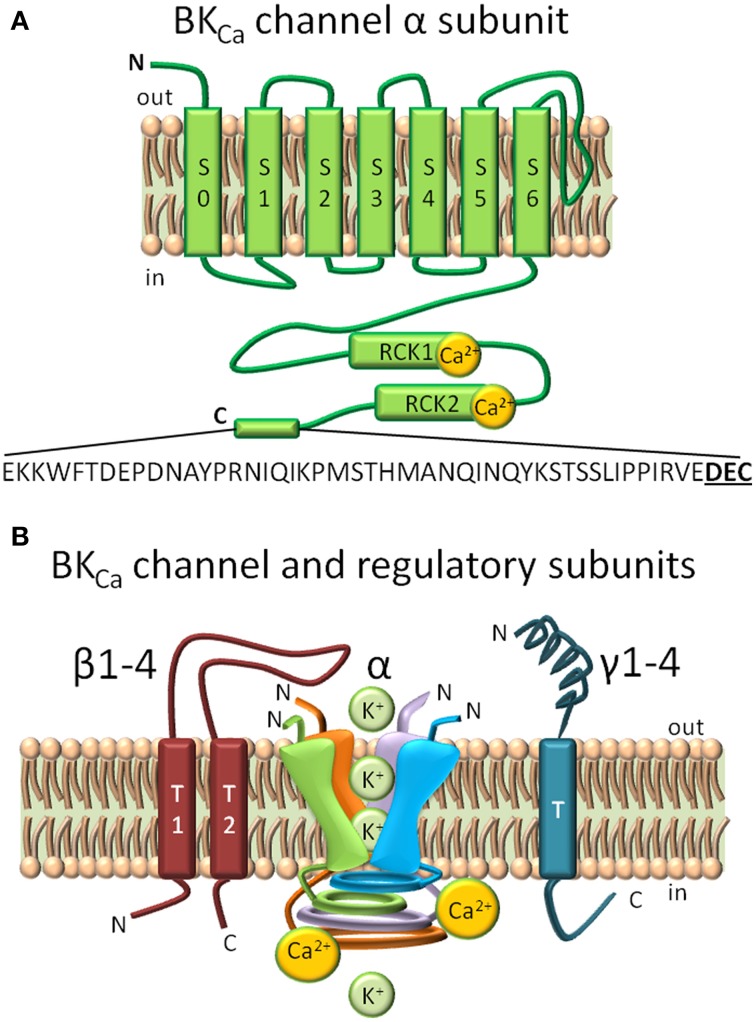
**Structural domains in BK_Ca_ channels and regulatory subunits**. **(A)** BK_Ca_ is composed by 7 transmembrane domains (S0–S7) and a long intracellular C-terminus. S0–S4 form the voltage sensing domain, and S5–S6 conform the pore-gating domain. Ca^2+^ biding sites are highlighted in the Regulator of Potassium Conductance (RCK) 1 and RCK2 domains. A C-terminal 50 amino acid splice insert, DEC, is highlighted. **(B)** Regulatory BK_Ca_ subunits. Homotetramer model of the pore-forming α subunit, the two spanning domain regulatory β subunits (1–4), and single spanning domain γ (1–4) subunits. The loop of β4 subunit confers to BK_Ca_ α subunit its resistance to toxin inhibition (Meera et al., 2000).

BK_Ca_ channels at the plasma membrane are characterized by having a large conductance, and by sensing changes in membrane potential and intracellular calcium (for a recent review see Contreras et al., 2013). Structure-function studies have ascribed these properties to distinct domains of the 7 transmembrane (S0–S7) α subunit -encoded by the *Kcnma1* gene- that has an extracellular N-terminus and an intracellular C-terminus (Figure 1A). Four α subunits form a functional channel. The voltage sensing domain encompasses S0–S4 segments, the pore/gate domain includes S5–S6 and corresponding linker which lines the pore selectivity filter of the tetrameric channel, and the Ca^2+^sensing domain is located at the C-terminus. Pore residues located extracellularly comprise the receptor for pore blockers, Charybdotoxin (ChTx) and Iberiotoxin (IbTx) (Gao and Garcia, 2003; Banerjee et al., 2013). The intracellular C-terminus, which occupies two thirds of the whole protein, contains two regions that can sense Ca^2+^ known as the regulators of K^+^ conductance (RCK) 1 and 2. Mutagenesis studies have shown that RCK1 contains two critical aspartates (D362/D367) while RCK2 contains 5 consecutive aspartates in the “Ca^2+^ bowl” that together are sufficient for BK_Ca_ activation at physiological Ca^2+^ concentrations (Schreiber and Salkoff, 1997; Xia et al., 2002). However, recent crystal structures have only detected a single site of Ca^2+^ binding located in the “Ca^2+^ bowl” and utilizing two main-chain carbonyl oxygens of Q889 and D892 and side-chain carboxylate oxygens of D895 and D897 (underlined in ^889^QFLDQDDDDDPDT^901^) (Yuan et al., 2010, 2012). In addition to Ca^2+^, BK_Ca_ can also be activated by Mg^2+^ in the millimolar range. Interestingly, residues of distinct α subunits form part of the Mg^2+^ sensor, namely D99 and N172 from the voltage sensing domain of one subunit with E374 and E399 from the RCK1 domain of a different subunit (Shi et al., 2002; Yang et al., 2008).

The *Kcnma1* gene when transcribed can undergo extensive alternative splicing that give rise to multiple BK_Ca_ channel isoforms with varied functional characteristics including voltage/Ca^2+^ sensitivities, response to phosphorylation and arachidonic acid modulation, and subcellular localizations, including targeting to mitochondria as discussed later in this review (Saito et al., 1997; Tian et al., 2001; Zarei et al., 2004; Ma et al., 2007; Li et al., 2010; Singh et al., 2013).

BK_Ca_ channel functional heterogeneity is further increased by its association with modulatory β (1–4) or γ (Yan and Aldrich, 2010, 2012) subunits (Figure 1B) that are mostly tissue-specific and greatly modify functional and pharmacological characteristics like kinetics, Ca^2+^/V sensitivities, and toxin blockade (Knaus et al., 1994; Wallner et al., 1999; Brenner et al., 2000; Meera et al., 2000; Uebele et al., 2000). Beta subunits (β1,β2,β4) can also act as modulators of channel density at the plasma membrane via endocytic processes (Toro et al., 2006; Zarei et al., 2007; Shruti et al., 2012; Cox et al., 2014). The relatively high tissue specificity of β subunits make them key in defining the function of BK_Ca_ channels in different organs, for example the abundance of β1 subunit in smooth muscle make them essential to maintain a healthy vascular tone.

Posttranslational modifications like lipidation and phosphorylation add another regulatory layer to BK_Ca_ function. For example, in the α subunit, internal myristoylation at N-terminal intracellular loops slows down the activation kinetics of BK_Ca_ channel and reduces its cell surface expression promoting endocytosis via a clathrin mediated mechanism (Alioua et al., 2011). Palmitoylation at S0–S1 linker also reduces cell surface expression, and at the spliced exon STREX (inserted at the C-terminus of BK_Ca_) produces channels resistant to protein kinase C induced inhibition (Jeffries et al., 2010; Zhou et al., 2012). With respect to β subunits, juxtamembrane palmitoylation of the β4 subunit at its C-terminus promotes surface expression of BK_Ca_ α subunits but only when the latter contain a 50 amino acid C-terminal splice insert, named DEC (Chen et al., 2013). Most of the above findings have been obtained in heterologous expression systems; the next challenge is to define the physiological or pathophysiological impact that these processes have in different organs of the body.

## Discovery of BK_Ca_ channel activity at the inner mitochondrial membrane and biophysical properties

The first evidence showing that BK_Ca_ channel with a conductance of about 300 pS (in 150 mM KCl) was present at the inner mitochondrial membrane was given by Siemen and coworkers in the late 90's; the channel was characterized using mitochondria devoid of external membranes (mitoplasts) of the glioma cell line, LN-229, and the patch clamp technique (Siemen et al., 1999). Since then, mitochondrial BK_Ca_ channels (mitoBK_Ca_) with similar conductances ranging from 200 to 307 pS have been detected in other systems using channel reconstitution in lipid bilayers or by patch clamping mitoplasts. A single report shows a 564 pS channel in mitochondria of brain (Table 1).

**Table 1:** **mitoBK_Ca_ biophysical properties in mammals**.

**Cell type/Organ/Method**	**Conductance, [K^+^], mM Pipette/bath or cis/trans**	**V_½_ or Open probability (Po) [Ca^2+^], μM**	**Ca^2+^, EC_50_**	**References**
Human glioma cell line (LN229) (on-mitoplast)	295 pS 150 K^+^/150 K^+^	At 8.7 Ca^2+^, V_½_ = −33 ±19 mV; At 1 Ca^2+^, V_½_ = 41 ± 23 mV	6.9 μM at −20 mV	Siemen et al., 1999
Guinea-pig ventricular myocytes (on-mitoplast)	307 pS 150 K^+^/150 K^+^	At 0.512 Ca^2+^, Po ~ 0.9 from −60 to +60 mV^*^	N/A	Xu et al., 2002
Rat ventricular myocytes (inside-out mitoplast)	270 pS 140 K^+^/140 K^+^	At 0.5 Ca^2+^, Po = 0.0087 at +40 mV	N/A	Ohya et al., 2005
Human glioma cell line (LN229 and LN405) (on-mitoplast)	276 pS 150 K^+^/150 K^+^	At 200 Ca^2+^, V_½_ ~ −42 mV^*^	N/A	Gu et al., 2007
Rat astrocytes (on-mitoplast)	295–296 pS 150 K^+^/150 K^+^	At 200 Ca^2+^, V_½_ ~ −50 mV^*^	N/A	Cheng et al., 2008, 2011
Human endothelial cell line (EA.hy926) (on-mitoplast)	270 pS 150/150 K^+^	At 100 Ca^2+^, V_½_ ~ +20 mV^*^	N/A	Bednarczyk et al., 2013b
Rat whole brain Lipid bilayers	265 pS 50 K^+^/450 K^+^	At 0 Ca^2+^, Po = 0.50 at +70 mV At 300 Ca^2+^, Po = 0.77 at +70 mV	N/A	Skalska et al., 2009
Rat whole brain Lipid bilayers	211 pS 200 K^+^/50 K^+^	At “contaminant” Ca^2+^, Po = 0.9 ± 0.01 at +40 mV, V_½_ = 11 ± 1 mV	N/A	Fahanik-Babaei et al., 2011a
Rat whole brain Lipid bilayers	565 pS 200 K^+^/50 K^+^	At 100 Ca^2+^, Po = 0.9 ± 0.05 at −40 to +40 mV. At 0 Ca^2+^, Po = 0.8 at +20 mV and Po = 0.07 at −40 mV	N/A	Fahanik-Babaei et al., 2011b

* *Estimated from published figure. On-mitoplast is also called mitoplast-attached configuration of the patch clamp technique. Abbreviations: V_½_, half activation potential or potential where an open probability of 0.5 is achieved; EC_50_, concentration of half maximal effect; N/A, not available*.

We now know that mitoBK_Ca_ and plasma membrane BK_Ca_ channel pore-forming α subunits are encoded by the same gene (*Kcnma1*) (Singh et al., 2013) explaining why they share common basic biophysical properties including a large conductance, and being responsive to voltage and Ca^2+^, although specific values may vary. A comparison of plasma membrane BK_Ca_ and mitoBK_Ca_ properties in a human glioma cell line (LN 229) shows that the conductance of the former was 199 ± 8 pS and of the latter was 278 ± 10 pS. Although both channels were voltage/Ca^2+^ dependent, their sensitivities were different. In the inside-out configuration, plasma membrane BK_Ca_ displayed a low sensitivity to voltage as it displayed a low open probability (Po) even at high potentials (Po < 0.1 at +80 mV and ~0.4 at 100 mV) and 400 μM Ca^2+^ facing the cytosolic side of the channel. mitoBK_Ca_, on the other hand, recorded on the on-mitoplast configuration and the same Ca^2+^ in the bath solution only needed a depolarization to −40 mV to reach a Po of ~0.6 (Gu et al., 2014). Although in this configuration the precise Ca^2+^ concentration at the matrix side of the channel is difficult to establish, the authors showed that by decreasing Ca^2+^ in the bath to “zero” the channel Po decreased to ≤0.05 (see below for discussion of mitoBK_Ca_ orientation). Thus, assuming that both classes of channels could sense the same Ca^2+^ concentration, one possible explanation to these differences is that multiple BK_Ca_ isoforms exist, both at the plasma membrane and in mitochondria, which could result from a combination of factors including splice variation, association with auxiliary subunits or posttranslational modifications affecting how they respond to voltage and Ca^2+^.

Along the above point of view, Table 1 shows that different cell types appear to express mitoBK_Ca_ channels with varied voltage and Ca^2+^ sensitivities. For example, the cardiac mitoBK_Ca_ channel from guinea pig had a particularly high Po of ~0.9 within a large voltage range (−60 to +60 mV) at 0.5 μM [Ca^2+^] suggesting that its molecular composition (e.g., association with auxiliary subunits) may be substantially distinct from that expressed in glioma mitochondria which at 1 μM Ca^2+^ displays a Po of 0.5 at +41 mV (half activation potential, V_1/2_ = 41 mV) (note that in both cases, experiments were performed in the on-mitoplast mode and Ca^2+^ was changed in the bath solution; thus, the exact Ca^2+^ concentration in the matrix side is unknown). This variability is not exclusive of mitoBK_Ca_ as different isoforms are also detected at the plasma membrane even within the same cell type. For example, BK_Ca_ channels from coronary smooth muscle can display a predominant V_½_ of −98 mV but also −66 mV, −38 mV, −16 mV and 21 mV (at 18 μM Ca^2+^) (Tanaka et al., 1997) that could be explained by α + β1 channels with different β1 subunit stoichiometry; while skeletal muscle BK_Ca_ single channels can have four-fold differences in K_0.5_ for Ca^2+^ with an average of 14 ± 7 μM at +30 mV (McManus and Magleby, 1991).

Obviously, a detailed biophysical and molecular characterization of mitoBK_Ca_is needed for each cell type to be able to understand the basis of their function.

## Orientation of mitoBK_Ca_; is the Ca^2+^ sensor facing the mitochondrial matrix?

Siemen et al. (1999) showed in on-mitoplast patches that increasing Ca^2+^ in the bath increased channel Po; the EC_50_ for Ca^2+^ measured at +60 mV was ~0.9 μM. The usage of a Ca^2+^ ionophore was dispensable, and thus, it was assumed that the normal Ca^2+^ import mitochondrial mechanisms were sufficient to increase matrix Ca^2+^ in the vicinity of mitoBK_Ca_ Ca^2+^sensor. In addition, mitoBK_Ca_ was blocked by ChTx (the toxin binds plasma membrane BK_Ca_ at the extracellular pore vestibule) applied to the patch pipette. ChTx displayed an EC_50_ of ~1.5 nM and a Hill coefficient of 1.2 consistent with the sensitivity reported for plasma membrane BK_Ca_ channels formed by α or α + β1 subunits (Meera et al., 2000). These results supported the idea that mitoBK_Ca_ ChTx receptor (in the pore vestibule) is facing the mitochondrial intermembrane space and thus, the Ca^2+^ sensor (located in the opposite side of BK_Ca_ protein) is facing the mitochondrial matrix.

Few years later Xu et al. (2002) investigated the presence of mitoBK_Ca_ in ventricular mitoplasts from guinea pig also using on-mitoplast patches and isotonic 150 mM KCl. Ventricular myocytes contained mitochondria rich in mitoBK_Ca_ whose conductance (~300 pS) was similar to that observed in the glioma cells (295 pS) (Table 1). Ventricular mitoBK_Ca_ activity was completely abolished by 200 nM ChTx applied to the patch pipette and the ensemble average patch currents augmented by increasing bath Ca^2+^ from 0.5 to 40 μM. In this case, the usage of a Ca^2+^ ionophore was also dispensable. Assuming that matrix Ca^2+^ was in equilibrium with the bath solution (aided by native Ca^2+^ import mechanisms), the results further supported the picture of mitoBK_Ca_ with its ChTx receptor facing the intermitochondrial space and thus, its Ca^2+^ sensor facing the matrix side.

Other studies have supported the orientation of mitoBK_Ca_ as being the pore vestibule facing the mitochondrial intermembrane space and the Ca^2+^ sensor in the matrix. Direct evidence using mitoplasts: (1) In the human glioma cell line LN-229, on-mitoplast patches showed an increased channel Po with matrix depolarization and exposing the external side of the mitoplast membrane to ChTx (using the whole-mitoplast or outside-out configurations and applying ChTx to the bath) reduced mitoBK_Ca_ activity (Gu et al., 2007, 2014); (2) In rat astrocyte mitoplasts, IbTx added to the pipette in the on-mitoplast configuration reduced mitoBK_Ca_ activity (Cheng et al., 2008), while depolarization of the inner side of the mitoplast (matrix side) promoted increased channel activity (Cheng et al., 2011). Indirect evidence using intact rat brain mitochondria: Ca^2+^-induced depolarization was prevented by IbTx (50 nM) and ChTx (200 nM) (Skalska et al., 2009). Because the outer mitochondrial membrane is permeable to proteins up to 5 kDa and IbTx as well as ChTx are ~4.2 kDa, both toxins are expected to traverse the outer membrane and reach the channel pore facing the intermembrane space; this arrangement would place the C-terminus facing the matrix.

The evidence supporting an opposite orientation, i.e., a C-terminus facing the intermembrane space is less clear. Recent recordings in inside-out mitoplast patches from a human astrocytoma cell line (U-87 MG) show a mitoBK_Ca_ channel that was activated by hyperpolarization of the matrix side (positive potentials applied to the pipette interior) and blocked by IbTx also applied to the matrix side (bath solution) (Bednarczyk et al., 2013a). Assuming that this channel is encoded by the *Kcnma1* gene (encoding plasma membrane BK_Ca_) (see below Singh et al., 2013), the above results would imply a pore vestibule facing the matrix and a C-terminus facing the intermembrane space. However, decreasing matrix Ca^2+^ decreased channel activity making the authors suggest that both IbTx and Ca^2+^ binding sites were probably facing the mitochondrial matrix. This hypothesis would support the existence of a mitoBK_Ca_ channel unrelated to the plasma membrane BK_Ca_, where the pore vestibule and the Ca^2+^ sensor are facing opposite sides of the plasma membrane (see Figure 1). It is evident that further studies on the orientation of mitoBK_Ca_ channel at the inner mitochondrial membrane are needed.

## mitoBK_Ca_ molecular origin

mitoBK_Ca_ molecular origin has been recently defined as the *Kcnma1* gene, which also encodes plasma membrane BK_Ca_ (Singh et al., 2013). Utilizing an exon-scanning RT-PCR strategy of the mouse *Kcnma1* gene and ventriculocyte mRNAs (these cardiac cells were an excellent system for this quest since they are characterized by their lack of BK_Ca_ channel activity at the plasma membrane) Singh et al., found transcript expression of 3 alternatively spliced exons, STREX, SV27 (27 amino acid insert) and the C-terminal 50 amino acid “DEC” exon. Notoriously, the amount of DEC exon transcripts equaled that of the total BK_Ca_ predicting a mitoBK_Ca_ tetrameric structure constituted by two subunits of BK_Ca_ variant containing DEC insert. Indeed, expression of BK_Ca_ constructs containing the DEC exon was sufficient for BK_Ca_ targeting to mitochondria in adult ventriculocytes. Mass spectrometry analysis and functional data using BK_Ca_ knockout mice further confirmed mitoBK_Ca_ as being encoded by the *Kcnma1* gene.

Interestingly, the ability of DEC exon for targeting mitoBK_Ca_ to mitochondria may vary depending on the cell type and/or its association with other subunits. In fact, expression in CHO cells of a BK_Ca_-DEC variant (containing additional alternatively spliced exons including SV27) cloned from hair cells showed prominent expression in Mitotracker labeled mitochondria but also at the cell periphery (Kathiresan et al., 2009). In contrast, expression of a DEC variant in COS-cells yielded signals confined to the endoplasmic reticulum (Ma et al., 2007). Thus, the DEC sequence may not be the only factor defining the targeting of BK_Ca_-DEC to mitochondria in all cell types but cell-specific mechanisms may exist that facilitate its mitochondrial delivery.

## mitoBK_Ca_ and regulatory subunits

BK_Ca_ regulatory β subunits are expressed in mitochondria from heart, skeletal muscle, endothelial cells and brain as discussed below. Whether BK_Ca_ γ subunits exist in mitochondria is still unknown.

In the heart, β1 subunit was found in mitoplasts isolated from rat ventricular myocytes and a two hybrid system showed its direct interaction with cytochrome c oxidase subunit I (Ohya et al., 2005). These results demonstrated the localization of β1 in the mitochondrial inner membrane and associated with a component of the respiratory chain. Furthermore, functional experiments showed that in rat ventricular mitoplasts mitoBK_Ca_ activity was enhanced by stimulation with 30 μM estradiol in the presence of 500 nM Ca^2+^ (Ohya et al., 2005). Under these conditions, estradiol is known to activate plasma membrane BK_Ca_ channels in complex with β 1 subunits (Valverde et al., 1999). Interestingly, in mitoplasts of astrocytes the stimulatory effect of 30 μM β-estradiol on mitoBK_Ca_ was transient resulting in a final inhibition of channel activity that was resilient to a second β-estradiol stimulus and was only partially restored by increasing Ca^2+^ in the matrix side of inside-out patches (Thiede et al., 2012).

In cultured pulmonary artery smooth muscle, β 1 subunit was found to play a role in 11,12-epoxyeicosatrienoic acid induced depolarization of mitochondrial membrane potential as this effect was abrogated in the β1^−/−^ animal (Loot et al., 2012).

In a human endothelial cell line, β2 was detected in lysates of mitochondria and mitoplasts. However, this subunit seems not to be forming complex with mitoBK_Ca_ in this cell line because the reported channel activity (Bednarczyk et al., 2013b) does not show classical time-dependent inactivation conferred by β2 (Wallner et al., 1999; Benzinger et al., 2006).

In rat soleus muscle, β4 is visualized both at the plasma membrane and in mitochondria using immunocytochemistry; and by immunoblotting as a protein of ~26 kDa using purified mitochondria (Skalska et al., 2008).

In the brain, β4 subunit is clearly localized to mitochondria of the gigantocellular reticular nucleus and in mitochondria of the pons. Because β 4 could not be proteolyzed in intact mitochondria but only after detergent solubilization, it was speculated that it must be internal to the outer mitochondrial membrane. Western blot analysis of brain homogenates and mitochondrial fractions further confirmed the presence of β4 in mitochondria. Interestingly, β2 was also found in brain mitochondrial fractions but not β1 nor β3 subunits (Piwonska et al., 2008). β4 has also been observed in mitochondria of rat hippocampal neurons by immunochemistry and as a protein of ~26 kDa in mitochondrial lysates of whole brain (Skalska et al., 2009). Thus, it is possible that β4 forms part of the mitoBK_Ca_ complex at the inner mitochondrial membrane of various types of neurons.

β4 subunit expression in the glia appears to be disease dependent as it was not found expressed in glia of adult normal brain (Piwonska et al., 2008) but it has been detected in mitochondria of a human gliobastoma cell line (U-87 MG) where it comigrates with cytochrome c oxidase subunit I (Bednarczyk et al., 2013a). Yet, the functional impact that β4 may have on mitoBK_Ca_ activity in glioma cells and in neurons needs to be established.

From the above studies it appears that mitochondrial β subunits show tissue specificity, a characteristic of their plasma membrane counterparts. However, more studies are needed to assess this hypothesis or to establish their functional role in mitochondria. Gene silencing models could be ideal for this task.

## mitoBK_Ca_ channel and protection from ischemic insult

mitoBK_Ca_channel was first related to cardiac protection from global ischemia and reperfusion injury by Xu et al. (2002) using a BK_Ca_ opener, NS1619 (10–30 μM). The drug used to precondition the heart prior ischemia and reperfusion, improved left ventricular developed pressure and decreased infarct size. Both effects were abolished with 1 μM Paxilline, an inhibitor of BK_Ca_. Several factors support the notion that NS1619 was opening BK_Ca_ located in mitochondria: (a) the fact that NS1619 could not be targeting plasma membrane BK_Ca_, as adult cardiomyocytes are known for their lack of sarcolemmal BK_Ca_ expression/activity (Singh et al., 2013; Schmitt et al., 2014); (b) mitochondrial K^+^ uptake was accelerated by NS1619 and decelerated by blocking BK_Ca_ with 100 nM IbTx; and (c) the protective effect of preconditioning the heart with NS1619 on reperfusion was not related to relaxation of the vasculature, where BK_Ca_ channel is abundant. Since then, other groups have confirmed and expanded these results (Table 2).

**Table 2:** **BK_Ca_ channel agonists and cardioprotection**.

**Model**	**Treatment**	**Baseline/Reperfusion (LVDP; mmHg)**	**Baseline/Reperfusion (CF; mL/min)**	**% Infarct size**	**References**
Guinea pig	Control	104 ± 9/34 ± 6	43 ± 6/18 ± 2	~50	Xu et al., 2002
	NS1619 (30 μM)	108 ± 3/**56 ± 5**	35 ± 5/19 ± 2	**~20**	
	NS+Paxilline	103 ± 14/33 ± 10	48 ± 3/21 ± 5	~55	
	Paxilline (1 μM)	113 ± 4/33 ± 5	51 ± 4/16 ± 1	~60	
Guinea pig	Control	ND	ND	~55	Stowe et al., 2006
	NS1619 (3 μM)			**~25**	
	NS1619 (3 μM) + Paxilline (1 μM)			~50	
	Paxilline (1 μM)			~55	
Infant rabbit	Control	51 ± 2	4 ± 1	14 ± 5	Shi et al., 2007
	NS1619 (10 μM)	**65 ± 4**	5 ± 1	**10 ± 5**	
	Paxilline (1 μM)	55 ± 10	5 ± 1	13 ± 3	
	Paxilline (pretreatment)	53.4 ± 9			
Rat	Control	~110/30 ± 3.3	ND	44.6±2	Bentzen et al., 2009
	Ischemic preconditioning	~120/**~65**		**7.9 ± 1.7**	
	NS11021 (1 μM)	~110/**60.3 ± 7.2**		**20.6 ± 4.5**	
	NS11021 (3 μM)	~110/**60.3 ± 7.2**		**11.4 ± 2**	
	NS 11021 (3 μM) + Paxilline (3 μM)	~110/~30		33.6 ± 5.6	
	NS11021 Postconditioning	~110/**~50**		**19.8 ± 3.3**	
Mouse	Control	~120/~60	ND	~50	Singh et al., 2013
	NS1619 (10 μM)	~120/**~120**		**~15**	

*Positive effects are highlighted in bold. Experiments used the isolated heart preparation. LVDP, Left Ventricular Developed Pressure; CF, Coronary flow; ND, not determined*.

Stowe et al. (2006) confirmed an improved left ventricular developed pressure by 3 μM NS1619 preconditioning and measured mitochondrial Ca^2+^ and ROS production. *In situ* recording of these parameters during the ischemia/reperfusion protocol (in the left ventricle of the isolated, perfused heart) demonstrated that both parameters decreased by NS1619 preconditioning both during the ischemia period and ~10 min after reperfusion started.

The cardioprotective effect of BK_Ca_ activation, in improving left ventricular developed pressure and reducing infarct size, has also been observed with BK_Ca_ opener NS11021 (1–3 μM) (Bentzen et al., 2009) and naringenin (4 μM) (Testai et al., 2013). Importantly, NS11021 is also effective when hearts or isolated cells are treated postischemia or postmetabolic inhibition and during reperfusion or re-energization, respectively (Bentzen et al., 2009; Borchert et al., 2013). These findings make BK_Ca_ an excellent target to improve cardiac function after an ischemic event as it occurs during heart infarct.

Interestingly, the protective effect of NS1619 may not be mediated by mitoBK_Ca_ in all systems like in primary rat cortical neurons. In this type of neurons, preconditioning with NS1619 (150 μM) caused mitochondrial depolarization (consistent with K^+^ influx) but this depolarization was not prevented by 5 min preincubation with 20 μM Paxilline (Gaspar et al., 2009). An alternative explanation to this negative result could be that Paxilline needs longer time to diffuse through the plasma membrane and reach mitoBK_Ca_ at the mitochondrial inner membrane.

Although BK_Ca_ channel activity is indeed inhibited by Paxilline (Zhou and Lingle, 2014), its exclusive use as pharmacological indicator of BK_Ca_ functional role in mitochondria or elsewhere needs to be taken with caution. This assertion is underscored by recent studies, where isoflurane preconditioning protected the heart from ischemic insult, showing that Paxilline (1 μM) abolished the anesthetic cardioprotective effect equally well in wild type and *Kcnma1*^−/−^ mice (Wojtovich et al., 2011, 2013).

It is also important to highlight that the specific action of NS1619 on BK_Ca_ channel activation has been questioned (Szewczyk et al., 2010). Concentrations of NS1619 above 10 μM inhibited SERCA with a consequent Ca^2+^ overload in sarcoplasmic reticulum and posterior cytosolic contamination (Wrzosek, 2014). Other authors also reported non-specific effects of NS1619 at concentrations ≥10 μM such as inhibition of mitochondrial respiratory chain (~15–30% inhibition at 10 μM) (Kicinska and Szewczyk, 2004; Cancherini et al., 2007), and H^+^/K^+^ leak with 50–100 μM of the drug (Aldakkak et al., 2010). Therefore, the use of low concentrations of NS1919 (i.e., ≤10 μM) in conjunction with the use of genetically modified models is desirable.

Conclusive evidence for the role of BK_Ca_ in cardioprotection has come from studies using BK_Ca_ knockout mouse models (*Kcnma1*^−/−^) whose hearts are not protected from ischemic injury by NS1619 (5–10 μM) or NS11021 (500 nM) (Singh et al., 2013; Wojtovich et al., 2013), or by ischemic preconditioning (Soltysinska et al., 2014) as revealed by measurements of heart function and infarct size in isolated perfused hearts. Mitochondrial BK_Ca_ of cardiomyocytes (Singh et al., 2013; Soltysinska et al., 2014) as well as BK_Ca_ expressed in cardiac neurons (Wojtovich et al., 2013) contribute to the cardioprotective effects. A role of BK_Ca_ expressed in cardiac neurons is supported by the fact that an inhibitor of neural transmission prevents protection by NS1619 (Wojtovich et al., 2013). Supporting a role for mitoBK_Ca_ are measurements in isolated mitochondria at 10 min of reperfusion showing improved Ca^2+^ retention capacity with NS1619 preconditioning a property that was absent in the knockout animal (Singh et al., 2013). In addition, a model of anoxia/reoxygenation of isolated mitochondria (mimicking the isolated heart ischemia/reperfusion model) showed that the cardioprotective effect of preconditioning could be related to a mitoBK_Ca_-mediated decrease of ROS production, as the production of ROS postanoxia was higher in the KO animal (Soltysinska et al., 2014).

## mitoBK_Ca_ and ROS production

The knockout animal studies described above point to mitoBK_Ca_ expression and ROS reduction as mitochondrial mechanisms playing a role in heart protection by ischemic preconditioning. The role of mitoBK_Ca_ activation in the regulation of ROS production has also been examined in heart mitochondria under basal conditions and has been found to depend on the ROS producing conditions. In succinate energized mitochondria, where ROS production at site I is enhanced by reversed electron flow, putative stimulation of mitoBK_Ca_ channel with 30 μM NS1619 produced a profound reduction of H_2_O_2_ production rate. Further, this decrease was partially overcome by pretreatment with 5 μM Paxilline (Heinen et al., 2007a). In contrast, when reverse electron flow was blocked by rotenone (i.e., succinate + rotenone), NS1619 increased H_2_O_2_ production rate and this effect was abolished by Paxilline (Heinen et al., 2007b). A decrease in ROS production by stimulating ROS production at complex I of the respiratory chain was also observed in brain mitochondria where activating mitoBK_Ca_ channel either with 10 μM CGS7184 or 3 μM NS1619 reduced H_2_O_2_ production mediated by malate-glutamate or succinate. The CGS7184-mediated decrease in ROS production was abolished by 50 nM IbTx supporting the involvement of mitoBK_Ca_ (Kulawiak et al., 2008). Experiments using the knockout models should provide definite proof of mitoBK_Ca_ role in the maintenance of normal levels of ROS to impede cell damage.

## mitoBK_Ca_ channel and mitochondrial transition pore (mPTP)

Under stress conditions, opening of mPTP allows the passage of small molecules with osmotic consequences for the cell like swelling and rupture of the mitochondrial outer membrane that permits liberation of apoptotic factors including cytochrome *c* causing cell death. The key elements responsible to keep mPTP closed are the mitochondrial Ca^2+^ content and ROS levels. Interestingly, mitoBK_Ca_ activity has been related to both ROS production as described above and to the regulation of mitochondrial Ca^2+^ content. Direct measurement of Ca^2+^ retention capacity (CRC) of cardiac mitochondria revealed that the protective effect of NS1619 preconditioning (increased CRC) was absent in the BK_Ca_ KO animal (Singh et al., 2013). These results imply that opening of BK_Ca_ to certain extent can protect mitochondria from uncontrolled mPTP opening. In line with this view, the proapoptotic protein Bax (Bcl-2 associated protein X) *directly* inhibits mitoBK_Ca_ activity in astrocyte mitoplasts but does not by itself produce mPTP electrical activity; and thus, Bax has been proposed to produce mPTP opening via inhibition of mitoBK_Ca_ (Cheng et al., 2011). In fact, inhibition of mitoBK_Ca_ with IbTx reduces the amount of Ca^2+^ necessary to depolarize brain mitochondria (a measure of increased mPTP activity) (Cheng et al., 2008) and increases cytochrome c release (from GL261 glioma mitochondria), a landmark of mPTP opening and apoptosis (Cheng et al., 2011).

From the above studies, it is evident that there is a physiological coupling between mitoBK_Ca_ and mPTP. Recent evidence indicates that mPTP is formed by dimers of the ATP-synthase (Giorgio et al., 2013). It would be interesting to test the hypothesis of a physical interaction between mitoBK_Ca_ and the ATP-synthase. The functional coupling of mitoBK_Ca_ with other components of the respiratory chain has been highlighted by the fact that mitoBK_Ca_ activity is affected by substrates of the respiratory chain in human astrocytoma U-87 MG cells (Bednarczyk et al., 2013a). The precise mechanisms of this coupling remain to be elucidated.

## Proposed mechanisms of mitoBK_Ca_ channel regulation of common pathological conditions

In mitochondria isolated from brain of diabetic rats, a K^+^ channel of 46 pS conductance that is inhibited by IbTx, a specific blocker of BK_Ca_, has been identified. This channel is voltage dependent with an effective valence of ~4.7 in contrast to ~2.6 of mitoBK_Ca_ from healthy brain (Noursadeghi et al., 2014). At present, it is difficult to ascribe a specific mechanism to this molecular switch. One possibility is that a subconductance of mitoBK_Ca_ becomes much more stable under diabetic conditions inasmuch a ~50 pS subconductance state of mitoBK_Ca_ has been reported in cardiac mitoplasts (Xu et al., 2002).

Cancerous tumor cells are highly resistant to hypoxia suggesting the presence of a mechanism(s) that prevents their death. mitoBK_Ca_ channels are expressed in cancerous cell lines (Siemen et al., 1999) and could participate in such mechanism as they are activated by hypoxia in mitoplasts derived from human glioma LN-229 cells and astrocytes (Gu et al., 2007, 2014; Cheng et al., 2008). Notably, hypoxia also reduces mPTP electrical activity in mitoplasts of liver mitochondria and delays mPTP opening in intact liver mitochondria as measured by Ca^2+^-induced membrane depolarization (Cheng et al., 2008). How would mitoBK_Ca_ sense oxygen levels during hypoxia? It is possible that chronic hypoxia-induced heme oxygenase-1 binds to the mitochondrial channel much like heme oxygenase-2 does with plasma membrane BK_Ca_ (Williams et al., 2004) promoting CO production and activation of mitoBK_Ca_.

Supporting the view that mitoBK_Ca_ may regulate mitochondrial function as a redox sensor is the fact that its electrical activity can be inhibited by 300 nM hemin, a byproduct of hemoglobin (Augustynek et al., 2014) with oxidative properties that increases drastically (~10 mM) during hemolysis like the one that occurs during hemorraghic stroke.

## Concluding remarks and perspectives

As a general conclusion, we can state that BK_Ca_ channel is present in the inner mitochondrial membrane of various cell types and in different species. The discovery that mitoBK_Ca_ is encoded by the same gene as the plasma membrane BK_Ca_ (*Kcnma1*) is just the beginning in our understanding of the molecular composition of mitoBK_Ca_ isoforms in different tissues that seem to display a spectrum of biophysical characteristics just like the plasma membrane channel. A detailed biophysical characterization in native mitochondria and in genetically engineered organelles should help in correlating distinct properties with molecular composition.

An immediate specific question to resolve is, for example, the orientation of mitoBK_Ca_ in the inner mitochondrial membrane. Experiments in inside-out patches where matrix and intermembrane spaces can have defined Ca^2+^ concentrations should solve this problem. Moreover, they will allow defining with certainty V_1/2_ and Ca^2+^ EC_50_ values for mitoBK_Ca_ in each cell type. At present, most of the experiments have been done in the on-mitoplast configuration and this information is lacking. Other points to address are: whether the BK_Ca_ γ subunit is present in mitochondria and if it forms part of mitoBK_Ca_ complex, or if β subunits have alternative functions in mitochondria.

Many other questions remain to be solved such as the physiological/pathophysiological role of mitoBK_Ca_ channel in different tissues, its subunit composition in different cell types, mechanisms of mitochondrial targeting, and interaction with mitochondrial protein complexes.

### Conflict of interest statement

The authors declare that the research was conducted in the absence of any commercial or financial relationships that could be construed as a potential conflict of interest.
